# Spinal Cord Signal Change on Magnetic Resonance Imaging May Predict Worse Clinical In- and Outpatient Outcomes in Patients with Spinal Cord Injury: A Prospective Multicenter Study in 459 Patients

**DOI:** 10.3390/jcm10204778

**Published:** 2021-10-18

**Authors:** Thorsten Jentzsch, David W. Cadotte, Jefferson R. Wilson, Fan Jiang, Jetan H. Badhiwala, Muhammad A. Akbar, Brett Rocos, Robert G. Grossman, Bizhan Aarabi, James S. Harrop, Michael G. Fehlings

**Affiliations:** 1 Division of Neurosurgery, Department of Surgery, University of Toronto, Toronto, ON M5T 2S8, Canada; thorsten.jentzsch@gmail.com (T.J.); jefferson.wilson@unityhealth.to (J.R.W.); fan.jiang@uhn.ca (F.J.); jetan.badhiwala@mail.utoronto.ca (J.H.B.); muhammad.akbar@mail.utoronto.ca (M.A.A.); brett.rocos@sickkids.ca (B.R.); 2 Division of Neurosurgery, Toronto Western Hospital, University Health Network, Toronto, ON M5G 2C4, Canada; 3 Department of Orthopedics, Balgrist University Hospital, University of Zurich, 8008 Zurich, Switzerland; 4 Division of Neurosurgery, Department of Clinical Neurosciences, University of Calgary Combined Spine Program, Hotchkiss Brain Institute, University of Calgary, Calgary, AB T2N 1N4, Canada; david.cadotte@ucalgary.ca; 5 Division of Neurosurgery, St. Michael’s Hospital, University Health Network, Toronto, ON M5T 2S8, Canada; 6 Department of Neurosurgery, Houston Methodist Hospital, Houston, TX 77030, USA; RGrossman@houstonmethodist.org; 7 Department of Neurosurgery, University of Maryland Medical Center and R Adams Cowley Shock Trauma Center, Baltimore, MD 21201, USA; BAarabi@som.umaryland.edu; 8 Departments of Neurological Surgery and Orthopedic Surgery, Thomas Jefferson University, Philadelphia, PA 19107, USA; james.harrop@jefferson.edu

**Keywords:** spinal cord injuries, magnetic resonance imaging, MRI, neurology, paralysis, walking, outcome

## Abstract

Prognostic factors for clinical outcome after spinal cord (SC) injury (SCI) are limited but important in patient management and education. There is a lack of evidence regarding magnetic resonance imaging (MRI) and clinical outcomes in SCI patients. Therefore, we aimed to investigate whether baseline MRI features predicted the clinical course of the disease. This study is an ancillary to the prospective North American Clinical Trials Network (NACTN) registry. Patients were enrolled from 2005–2017. MRI within 72 h of injury and a minimum follow-up of one year were available for 459 patients. Patients with American Spinal Injury Association impairment scale (AIS) E were excluded. Patients were grouped into those with (*n* = 354) versus without (*n* = 105) SC signal change on MRI T2-weighted images. Logistic regression analysis adjusted for commonly known a priori confounders (age and baseline AIS). Main outcomes and measures: The primary outcome was any adverse event. Secondary outcomes were AIS at the baseline and final follow-up, length of hospital stay (LOS), and mortality. A regression model adjusted for age and baseline AIS. Patients with intrinsic SC signal change were younger (46.0 (interquartile range (IQR) 29.0 vs. 50.0 (IQR 20.5) years, *p* = 0.039). There were no significant differences in the other baseline variables, gender, body mass index, comorbidities, and injury location. There were more adverse events in patients with SC signal change (230 (65.0%) vs. 47 (44.8%), *p* < 0.001; odds ratio (OR) = 2.09 (95% confidence interval (CI) 1.31–3.35), *p* = 0.002). The most common adverse event was cardiopulmonary (186 (40.5%)). Patients were less likely to be in the AIS D category with SC signal change at baseline (OR = 0.45 (95% CI 0.28–0.72), *p* = 0.001) and in the AIS D or E category at the final follow-up (OR = 0.36 (95% CI 0.16–0.82), *p* = 0.015). The length of stay was longer in patients with SC signal change (13.0 (IQR 17.0) vs. 11.0 (IQR 14.0), *p* = 0.049). There was no difference between the groups in mortality (11 (3.2%) vs. 4 (3.9%)). MRI SC signal change may predict adverse events and overall LOS in the SCI population. If present, patients are more likely to have a worse baseline clinical presentation (i.e., AIS) and in- or outpatient clinical outcome after one year. Patients with SC signal change may benefit from earlier, more aggressive treatment strategies and need to be educated about an unfavorable prognosis.

## Key Points

1. This is a report on a prospective registry with an exceptionally large sample size of 459 traumatic spinal cord injury patients and a long follow-up for trauma populations;

2. Spinal cord (SC) signal change on initial magnetic resonance imaging may be an independent predictor of adverse events after controlling for age and baseline neurological impairment (odds ratio of 2.09 for adverse events; odds ratios of 0.45 and 0.36 for ambulation at baseline and final follow-up, respectively);

3. Patients with SC signal change may benefit from earlier, more aggressive treatment strategies and need to be educated about an unfavorable prognosis.

## 1. Introduction

Spinal cord (SC) injury (SCI) is a devastating event and risk factors for the clinical outcome play an important role in patient management and education. The prevalence of traumatic SCI ranges from 236–1009 per million [1], but is likely underestimated due to a high mortality rate at the time of injury and limited diagnosis. One of the highest incidences of SCI is found in the United States, with 54 cases per million per year [2], while low rates have been reported for Spain with 8 cases per million population [3]. The levels of injury vary and incomplete tetraplegia (34%) is more common than complete paraplegia (25%), complete tetraplegia (22%), and incomplete paraplegia (17%) [4].

Physicians struggle with providing optimal care, defining the resources they need, and explaining the prognosis due to limited available data. Although magnetic resonance imaging (MRI) is a powerful imaging modality, there has been a lack of evidence regarding prospective MRI assessment and potential clinical outcomes [5,6,7,8,9,10,11,12,13,14,15,16,17,18]. A limited number of studies have reported on the association between SC intraparenchymal signal change and the clinical outcome [5,6,7,8,11,13,14,15,16,17,18,19], but these studies have been limited by small sample sizes with heterogeneous populations. Further, these reports also lacked long-term follow-up [5,10], inclusion of injuries to the entire spine [6], patients with an ossified posterior longitudinal ligament (OPLL) [7,8,14], SCI without radiographic abnormality (SCIWORA) [11], upper extremity impairment [16], and postoperative imaging assessment [19].

Therefore, this study aimed to investigate whether baseline MRI features predicted the clinical course of the disease. To definitively understand this relationship, a large, prospective patient cohort with SCI was examined. Based on preliminary understandings of SCI and radiological findings, it was hypothesized that MRI-assessed SC signal change at baseline would be a predictor for increased in- and outpatient adverse events and worse functional outcomes.

## 2. Materials and Methods

This is a study based on data from the prospective North American Clinical Trials Network (NACTN) registry [20,21], with prospective collection of imaging data and pre-determined clinical endpoints after ethical approval (CAPCR-ID: 05-0626) and with informed consent. NACTN, established in 2004, is a consortium of international neurosurgical institutions. The database is registered on ClinicalTrials.gov (accessed on 13 October 2021) [22]. NACTN’s goals are to organize a multicenter network and provide a large database with which to study and improve the course of disease and adverse events of SCI.

Patients were enrolled into the NACTN database from June 2005 to March 2017. Neurologically intact patients and those with American Spinal Injury Association impairment scale (AIS) E were excluded. Patients were included if MRI was performed within 72 h of the SCI and a minimum clinical follow-up evaluation at one year was available, of which 459 patients met these criteria. Patients were grouped into those with (*n* = 354) versus (vs.) without (*n* = 105) SC signal change detected on their MRI immediately after injury. MRI SC signal change was defined as sagittal and/or axial T2-weighted signal change, read by trained radiologists and entered into the database by each participating site [23]. The MRI scanner type and field strength varied depending on the institution. SC signal change was chosen as it is thought to represent injury to the SC, which likely has implications for clinical function.

The primary outcome was the presence of one or more adverse events. The definition of an adverse event was that offered by Jiang et al. [24], including all adverse events recorded by the participating centers consisting of cardiopulmonary, pulmonary embolus, deep vein thrombosis, gastrointestinal and genitourinary, hematologic, skin, systemic infection, urinary tract infection, wound infection, neurological deterioration, hardware failure, and other (unspecified) adverse events.

The secondary outcome measures were: baseline and final AIS at the last follow-up [4], length of hospital stay, and mortality.

An extensive literature search was also undertaken for previous literature on prospective studies about acute SCI, MRI, and complications (Table 1. PubMed.gov was searched with the terms “prospective, acute spinal cord injury, magnetic resonance imaging, complications”.

Data are given in absolute numbers with percentages (%) and medians with interquartile ranges (IQRs). For univariate analysis, the Wilcoxon rank sum and chi-squared tests were used. For multivariate analysis, logistic regression models were chosen due to the categorical nature of the data. AIS was categorized into AIS D (ambulatory) vs. AIS A-C (non-ambulatory). The analysis adjusted for commonly known a priori confounders (instead of a preliminary analysis for predictor identification), age, and baseline AIS, and included 435 patients due to missing data in the age category (*n* = 24). Surgery was not included in the analysis since this would have reduced the patient number even further. Of note, even when including this variable, the results did not change substantially. The performance of the model was acceptable according to the goodness-of-fit test by Hosmer-Lemeshow. We also calculated the sensitivity, specificity, positive predictive value (PPV), negative predictive value (NPV), likelihood ratios (LRs) and area under the curve. *p*-values < 5% were considered significant. In a post hoc power calculation, the power was 96.0% (considering the adverse events in each SC signal change group (65.0% (*n* = 354) vs. 44.8% (*n* = 105)) and an alpha of 5.0%). Analyses were carried out using Stata (version IC 13.1; StataCorp LP, College Station, TX, USA).

## 3. Results

SCI traumatic patients with MRI SC signal change were younger (46.0 (interquartile range (IQR)) 29.0 vs. 50.0 (IQR 20.5) years, *p* = 0.039). There were no differences in the other baseline variables, i.e., gender (females: 65 (19.1%) vs. 21 (20.0%), *p* = 0.831), body mass index (25.9 (IQR 5.9) vs. 25.8 (7.0), *p* = 0.708), smoking (95 (27.8%) vs. 20 (19.6%), *p* = 0.098), comorbidities (138 (40.1%) vs. 42 (40.0%), *p* = 0.983), mechanism of injury (fall: 132 (39.1%) vs. 49 (49.5%), *p* = 0.116), and injury location (cervical: 273 (78.5%) vs. 88 (84.6%), *p* = 0.388) (Table 2).

Adverse events were observed in 277 (60.4%) patients. The most common adverse event was cardiopulmonary (186 (40.5%)). There were more adverse events in patients with SC signal change (230 (65.0%) vs. 47 (44.8%), *p* < 0.001) (Table 3). These differences remained significant in a logistic regression model (odds ratio (OR) = 2.09 (95% confidence interval (CI) 1.32–3.35), *p* = 0.002), indicating that patients with SC signal change were 109% more likely to develop an adverse event than patients without SC signal change, when controlling for other factors (Table 4). The sensitivity of the SC signal change for adverse events was 83.0% and the specificity was 31.9%. The PPV was 64.9% and the NPV was 55.2%. The positive LR was 1.22 (95% CI 1.09–1.36) and the negative LR was 0.53 (0.38–0.75). The area under the curve was 0.57 (95% CI 0.53–0.62).

Patients with SC signal change at baseline had significantly worse neurologic injuries (OR = 0.45 (95% CI 0.28–0.72), *p* = 0.001) and final follow-ups (OR = 0.36 (95% CI 0.16–0.82), *p* = 0.015) when adjusting for age (OR = 1.00 (95% CI 0.99–1.02), *p* = 0.379 and OR = 1.03 (95% CI 1.01–1.05), *p* < 0.001, respectively). This indicated that patients with SC signal change were 55% and 64% less likely to be in the AIS D category at baseline and AIS D or E category at final follow-up after one year, respectively, than patients without SC signal change.

The length of stay was significantly longer in patients with SC signal change (13 (IQR 17.0) vs. 11 (IQR 14.0), *p* = 0.049). There was no difference in mortality (11 (3.2%) vs. 4 (3.9%), *p* = 0.767) (Table 2).

The results of the literature search on prospective studies about acute SCI, MRI, and complications are shown in Table 5 and Table S1 in Supplementary Material.

## 4. Discussion

### 4.1. Main Findings

This ancillary study on the prospective NACTN registry [20] reviewed 459 patients with a relatively long follow-up in a trauma population of at least one year. The data showed that SC signal change on the initial MRI after traumatic SCI is an independent predictor of adverse events, as defined by Jiang et al. [24]. This factor remained significant even after controlling for age and baseline AIS impairment, and patients with SC signal change were 109% more likely to suffer an adverse event. SC signal change is consistent with significant neurologic impairment in that 55% were ambulatory at baseline and were 64% less likely to be ambulatory at the final follow-up. The length of stay in the hospital was also longer in patients with SC signal change, but there was no difference in mortality. The SC signal intensity appears to be a rapid and accurate method with which to assess the predicted outcome, tailor the treatment options (e.g., early surgery), and educate the patient, so as to potentially alter the actual outcome.

The initial mechanical force acting on the SC is the primary injury. These injuries are mostly due to impact with persistent compression (e.g., bone fracture fragments) but can also be due to impact with transient compression (e.g., hyperextension injury). These forces damage the SC pathways and blood vessels, which leads to secondary injuries by several mechanisms, such as vascular malfunctioning (acute phase), Wallerian degeneration (subacute phase), and glial scarring (chronic phase), as summarized by Alizadeh et al. [54]. In the authors’ opinion, it is very important to obtain and carefully assess initial MRIs after traumatic SCI to make general predictions of the patient’s immediate and long-term outcome.

### 4.2. Current Knowledge and Addition of Our Findings

The previous literature on the predictive nature of SC signal change for the clinical baseline and outcome in patients with SCI is sparse. A detailed literature search of SCI and MRI signal change was performed with a systematic review [5,6,7,8,9,10,11,12,13,14,15,16,17,18,25,26,27,28,29,30,31,32,33,34,35,36,37,38,39,40,41,42,43,44,45,46,47,48,49,50,51] (Table 1 and Table 5). Fourteen studies met the inclusion criteria, as defined as being prospective and examining acute SCI, MRI, and adverse events [5,6,7,8,9,10,11,12,13,14,15,16,17,18]. Aside from the limited number of studies and their lack of controlling for confounders such as age [5,6,7,8,9,10,11,12,13,14,15,16,17,18], the available reports are limited by short-term follow-up (5, 10), a smaller sample size [9,12,13,15,17,18], cervical spine only [6], OPLL [7,8,14], SCIWORA [11], and upper extremity impairment [16].

Herein, the previous literature on associations between imaging findings and clinical outcomes is described. Rutges et al. [5] investigated the MRI signal change during the first three postoperative weeks (*n* = 19). They reported that the SC T2 signal increased within the first 48 h but decreased thereafter. Martínez-Pérez et al. [6] described the radiologic findings for the neurological prognosis (*n* = 86). They noted that a T2 signal > 36 millimeters (mm) and facet dislocation predicted a worse neurological outcome. Gu et al. [7] and Kwon et al. [8] studied radiological outcome predictors in SCI patients with ossified posterior longitudinal ligament (OPLL) (*n* = 36 and *n* = 38, respectively). The authors reported that high-intensity zones and a higher intramedullary signal intensity grade, as well as space available for the cord, were associated with worse outcomes. Freund et al. [9] investigated neuronal degeneration above the SCI lesion level (*n* = 13 and 18 controls). The authors found a rapid decline in cross-sectional spinal cord area and an association between decreased cross-sectional SC loss and improved SC independence. Maeda et al. [10] reported extraneural soft-tissue damage and clinical relevance in patients without bone injury (*n* = 88). They showed an association between anterior longitudinal disruption, disc damage, and prevertebral hyperintensity with AIS motor scores. Machino et al. [11] reported the occurrence rate of increased signal intensity (ISI) and prevertebral hyperintensity (PVH) in patients with cervical SCI without radiographic abnormality (SCIWORA) (*n* = 100). They found that ISI and PVH were common (92% and 90%, respectively), particularly in AIS A-B patients (100% each), and noted a negative correlation between the ISI and preoperative Japanese Orthopedic Association (JOA) score as well as its recovery rate. Miyanji et al. [12] studied MRI associations with the neurologic status (*n* = 100). These authors reported that the maximum SC compression and lesion length were associated with complete motor and sensory SCI. Further, they noted that edema, hemorrhage, cord swelling, stenosis, and soft-tissue injury were associated with complete SCI. Boldin et al. [13] investigated SCI hemorrhage and the length of hematoma as predictors of recovery (*n* = 29). They reported a hemorrhage >4 mm to be associated with complete injury. The edema and hematoma length were also longer in AIS A patients. Koyanagi et al. [14] investigated radiographic and clinical findings in patients with OPLL. Their results showed intramedullary hyperintensity and paravertebral soft tissue injuries in all four patients with Frankel grades A and B, in 80% with Frankel C, and 56% in Frankel D. Takahashi et al. [15] studied the association between image findings and clinical outcomes (*n* = 43). They reported that a baseline low-intensity T2 signal was associated with a poor prognosis. A high-intensity signal after two to three weeks was also associated with permanent paralysis. Ishida and Tominaga [16] evaluated MRI predictors for neurological recovery in patients with only upper-extremity impairment (*n* = 22). Their results showed that an absence of abnormal signal intensity was the best predictor of neurological recovery. Koyanagi et al. [17] reported on MRI predictors of the worse outcomes in patients without a fracture or dislocation (*n* = 42). Intramedullary hyperintensity on T2-weighted images was associated with more severe neurological deficits. Shimada and Tokioka [18] investigated MRI findings and clinical outcomes. Their results showed that T2-weighted images were associated with the severity of spinal cord damage and clinical outcome.

This study is limited by the inherent issues with registries and the heterogeneity of the SCI population. Since multiple institutions are involved, the exact timing of the MRI, the type and setting of the MRI scanner, and the availability and validity of data regarding the clinical assessments may vary. Future studies may add other potential predictor variables, such as corticosteroid use and comorbidities in their regression models. Although we controlled for age in the final regression model and there were no statistical differences in the mechanism of injury, the cohort with SC signal change was younger and included more motor vehicle accidents. Future studies should focus on this issue. Subsequent studies may also investigate different MRI findings, such as tissue bridges [55], the benefit of early surgical intervention in patients with SC signal change, and the use and prediction of subsequent MRIs and longer follow-ups. Another limitation is that many adverse events were not specified in detail, thus limiting further sub-analysis or assessment of the actual severity of adverse events. Future studies should focus on specifying adverse events in as much detail as possible. Furthermore, cervical SCI accounts for almost 80% of cases in this study, while the number of thoracic SCI cases was low. This constitutes a substantial limitation of this study, which analyzed the entire SCI spectrum, based on this composition. Future studies could opt to include more thoracic SCI cases.

## 5. Conclusions

MRI SC signal change may predict the clinical course of disease in patients after acute traumatic SCI. If signal change is present, patients are more likely to have a lower baseline clinical presentation as well as a decreased in- or outpatient clinical outcome after one year. Therefore, patients with SC signal change may benefit from earlier and more aggressive treatment strategies and need to be educated about an unfavorable prognosis.

## Figures and Tables

**Table 1:** Excluded studies of previous literature on prospective studies about acute spinal cord injury, magnetic resonance imaging, and complications (*n* = 41) [25,26,27,28,29,30,31,32,33,34,35,36,37,38,39,40,41,42,43,44,45,46,47,48,49,50,51].

Exclusion Criterion	Studies (*n*)
No acute SCI	9 [25,26,27,28,29,30,31,32,33]
No MRI of the spine	6 [34,35,36,37,38,39]
Experimental study	4 [40,41,42,43]
Case report	2 [44,45]
Focus on brain injury	2 [46,47]
Heterogenous study population (not exclusively SCI patients)	1 [48]
Metastatic SC compression	1 [49]
Neurological condition (amyotrophic lateral sclerosis)	1 [50]
No association between imaging and clinical outcome	1 [51]

Note: PubMed.gov (accessed on 31 August 2020) search with the terms “prospective, acute spinal cord injury, magnetic resonance imaging, complications”.

**Table 2:** Baseline data for spinal cord injury patients stratified by radiographic spinal cord signal change (*n* = 459).

		Spinal Cord T2 Signal Change				
		Yes (*n* = 355)		No (*n* = 105)		
Variable		Median	(IQR)	Median	(IQR)	*p*-Value *
Age (*n* = 435)		46.0	(29.0)	50.0	(20.5)	0.039
Gender (n = 446), *n* (%)						0.831
	Female	65	(19.1)	21	(20.0)	
	Male	277	(81.0)	84	(80.0)	
BMI (*n* = 422)		25.9	(5.9)	25.8	(7.0)	0.708
Smoker (*n* = 444), *n* (%)		95	(27.8)	20	(19.6)	0.098
Comorbidities (*n* = 449), *n* (%)		138	(40.1)	42	(40.0)	0.983
Mechanism of injury (*n* = 437), *n* (%)						0.116
	Fall	132	(39.1)	49	(49.5)	
	Motor vehicle accident	154	(45.4)	34	(34.3)	
	Sports	32	(9.5)	7	(7.1)	
	Other (assault, blast)	20	(5.9)	9	(9.1)	
Injury location (*n* = 452), *n* (%)						0.169
	Cervical	273	(78.5)	88	(84.6)	
	Thoracic and lumbosacral conus	75	(21.5)	16	(15.4)	

* Wilcoxon rank sum or chi-squared test. Abbreviations: n (absolute number); IQR (interquartile range); % (percent); BMI (body mass index).

**Table 3:** Clinical outcome data for spinal cord injury patients stratified by radiographic spinal cord signal change (*n* = 459).

		Spinal Cord T2 Signal Change				
		Yes (*n* = 354)		No (*n* = 105)		
Variable		Median	(IQR)	Median	(IQR)	*p*-Value *
Adverse events, *n* (%) ^†^		230	(65.0)	47	(44.8)	<0.001
	Cardiopulmonary	157	(68.3)	29	(61.7)	0.383
	Pulmonary embolus	12	(5.2)	2	(4.3)	0.784
	DVT	16	(7.0)	2	(4.3)	0.494
	Systemic	11	(4.8)	5	(10.6)	0.117
	UTI	45	(19.6)	7	(14.9)	0.455
	GI and GU	28	(12.2)	5	(10.6)	0.767
	Wound infection	5	(2.2)	3	(6.4)	0.116
	Hematology	78	(33.9)	16	(34.0)	0.986
	Skin	35	(15.2)	6	(12.7)	0.666
	Neurological	60	(26.1)	12	(25.5)	0.937
	Hardware failure	3	(1.3)	3	(1.3)	0.431
	Other (unspecified)	143	(62.2)	22	(46.8)	0.050
AIS D						
	Baseline	175	(49.7)	72	(68.6)	0.001
AIS D or E						
	Follow-up	288	(81.1)	97	(92.4)	0.006
Length of stay (*n* = 442)		13.0	(17.0)	11.0	(14.0)	0.049
Death (*n* = 443), *n* (%)		11	(3.2)	4	(3.9)	0.767

* Wilcoxon rank sum or chi-squared test; ^†^ It was possible that patients experienced more than one adverse event, and other (unspecified) refers to adverse events that were not further specified. Abbreviations: *n* (absolute number); IQR (interquartile range); % (percent); DVT (deep vein thrombosis); UTI (urinary tract infection); GI (gastrointestinal); GU (genitourinary), AIS (American Spinal Injury Association impairment scale).

**Table 4:** Logistic regression model for adverse events in spinal cord injury patients (*n* = 435).

Variable	OR	95% CI	*p*-Value *
Spinal cord T2 signal change	2.09	(1.31–3.35)	0.002
Age	1.00	(0.99–1.01)	0.598
AIS D at baseline	0.36	(0.24–0.55)	<0.001

* Wald test. Note: This logistic regression model included all shown variables (adverse events, spinal cord T2 signal change, age, and AIS D at baseline) (pseudo R2 = 0.066). The analysis adjusted for commonly known a priori confounders (instead of a preliminary analysis for predictor identification), age, and AIS D at baseline. Age and AIS D were chosen as they are known to influence the outcome (e.g., younger patients and AIS D (i.e., ambulatory) patients are less likely to have an unfavorable outcome after spinal cord injury compared to elderly patients and AIS A-C (non-ambulatory) patients) [52,53]. Importantly, the other potential predictors (Table 2) did not show any statistical associations in the univariate analysis, confirming our choice of a priori confounders. Abbreviations: OR (odds ratio); % (percent); CI (confidence interval); AIS (American Spinal Injury Association impairment scale).

**Table 5:** Previous literature on prospective studies about acute spinal cord injury, magnetic resonance imaging, and adverse events (*n* = 41) [5,6,7,8,9,10,11,12,13,14,15,16,17,18].

Study Number	Author(s)	Patients (n)	Aim	Main Finding	Age		Limitation
					Mean	(SD)	
1	Rutges et al. [5]	19	Change during first three postoperative weeks	SC edema length increased within first 48 h, but decreased thereafter Hematoma in all AIS-A and B patients	57.2	(15.1)	Short-term follow-up (three weeks)
2	Martínez-Pérez et al. [6]	86	Radiologic findings for neurologic prognosis	Edema > 36 mm and facet dislocation predicted worse neurological outcome	47.6	na	Limited patient number and cervical spine only
3	Gu et al. [7]	36	Outcome predictors in patients with OPLL	High-intensity zones (vs low-intensity zones) were associated with worse outcomes (mJOA improvement of 2.5 (SD 2.8) vs. 6.3 (1.5) points)	53.5	(13.3)	Limited to OPLL
4	Kwon et al. [8]	38	Outcome predictors in patients with OPLL	Higher intramedullary signal intensity grade and space available for cord were associated with worse outcomes	62.7	na	Limited to OPLL
5	Freund et al. [9]	13 (18 controls)	Neuronal degeneration above the lesion level	Rapid decline in cross-sectional spinal cord area Decreased cross-sectional SC loss was associated with improved SCIM scores	46.9	(20.2)	Limited patient number
6	Maeda et al. [10]	88	Extraneural soft-tissue damage and clinical relevance in patients without bone injury	Association between anterior longitudinal disruption, disc damage, and prevertebral hyperintensity with AIS motor score	64	na	Short-follow-up (mean six months (range of one to seven months))
7	Machino et al. [11]	100	Occurrence rate of ISI and PVH in patients with cervical SCIWORA	ISI and PVH in 92% and 90%, respectively ISI and PVH in 100% each in AIS A and B patients. Negative correlation between ISI and preoperative JOA score and recovery rate of JOA score	55	na	Limited to SCIWORA
8	Miyanji et al. [12]	100	MRI association with neurologic status	MSCC and lesion length was associated with complete motor and sensory SCI Edema, hemorrhage, cord swelling, stenosis, and soft-tissue injury associated with complete SCI MSCC and MCC correlated with baseline AIS motor scores MSCC, cord swelling, and hemorrhage predictive of neurological outcome Cord swelling and hemorrhage correlated with AIS score after controlling for baseline neurologic assessment	45	na	Limited number of patients
9	Boldin et al. [13]	29	Investigated spinal cord hemorrhage and length of hematoma as predictors of recovery	Hemorrhage > 4 mm was associated with complete injury Edema and hematoma length were longer in AIS A patients	43.5	18.1	Limited patient number
10	Koyanagi et al. [14]	28	Radiographic and clinical findings in patients with OPLL	Intramedullary hyperintensity and paravertebral soft tissue injuries in all four patients with Frankel grades A and B, in 80% with Frankel C, and 56% in Frankel D Paravertebral soft tissue injuries were also associated with Frankel grades A–C	63.0	na	Limited to OPLL
11	Takahashi et al. [15]	43	Investigated association between image findings and clinical outcome	Baseline low-intensity T2 signal was associated with poor prognosis High-intensity signal after 2–3 weeks was associated with permanent paralysis	63.4	na	Limited patient number
12	Ishida and Tominaga [16]	22	Evaluated MRI predictors for good neurologic recovery in patients with only upper extremity impairment	Absence of abnormal signal intensity was best predictor of neurologic recovery	45.9	na	Limited patient number
13	Koyanagi et al. [17]	42	MRI predictors of worse outcome in patients without fracture or dislocation	Intramedullary hyperintensity on T2-weighted images was associated with more severe neurological deficits	58.9	na	No results on association between MRI and clinical outcome
14	Shimada and Tokioka [18]	75	MRI findings and clinical outcomes	T2-weighted images were associated with severity of spinal cord damage and clinical outcome Best time for imaging is at time of injury and two to three weeks later	54.7	na	Limited patient number

Note 1: PubMed.gov (accessed on 31 August 2020) search with the terms “prospective, acute spinal cord injury, magnetic resonance imaging, adverse events”; Note 2: The variables used for MRI and clinical outcome evaluation are heterogenous. Previous studies most commonly described SC edema [5,6,13] (“signal intensity” [7,8,11,14,15,16,17,18]) in their MRI evaluation, but also hematoma [5,13], space available for cord [8], cross-sectional SC area [9], anterior longitudinal disruption [10], disc damage [10], prevertebral hyperintensity [10,11], maximum spinal cord compression [12], lesion length [12], cord swelling [12], stenosis [12], soft-tissue injury [12], and maximum canal compromise [12]. They often focused on the AIS grade [5,6,13,18] in their clinical evaluation, but also mentioned the mJOA [7] and JOA [11], AIS motor score [8,10,12,16], spinal cord independence measure [9], Frankel grade [14], incomplete and complete paralysis [15,17]. Abbreviations: *n* (absolute number); SD (standard deviation); SC (spinal cord); h (hours); AIS (American Spinal Injury Association impairment scale); mm (millimeters); na (not applicable); vs. (versus); OPLL (ossification of posterior longitudinal ligament); mJOA (modified Japanese Orthopedic Association); SCIM (spinal cord independence measure); ISI (increased signal intensity); PVH (prevertebral hyperintensity); SCIWORA (spinal cord injury without radiographic abnormality); MRI (magnetic resonance imaging); MSCC (maximum spinal cord compression); MCC (maximum canal compromise).

## Data Availability

All data is reported in this study.

## Supplementary Materials

The following are available online at https://www.mdpi.com/article/10.3390/jcm10204778/s1, Table S1: Excluded studies of previous literature on prospective studies about acute spinal cord injury, magnetic resonance imaging, and complications (*n* = 41), References S1: Supplementary references.
