# Supplementary material for: Spinal Cord Signal Change on Magnetic Resonance Imaging May Predict Worse Clinical In- and Outpatient Outcomes in Patients with Spinal Cord Injury: A Prospective Multicenter Study in 459 Patients
Source: J Clin Med. 2021 Oct 18;10(20):4778. doi: 10.3390/jcm10204778 (PMC8537526; doi:10.3390/jcm10204778)
Supplement: Supplementary file 1 [file jcm-10-04778-s001.zip › jcm-1322085-supplementary.pdf]

SUPPLEMENTARY MATERIALS

Table S1.

Excluded studies of previous literature on prospective studies about acute spinal cord injury, magnetic resonance imaging, and complications (n=41) [1-27].

Exclusion criterion	Studies (n)
No acute SCI	9[1-9]
No MRI of the spine	6[10-15]
Experimental study	4[16-19]
Case report	2[20, 21]
Focus on brain injury	2[22, 23]
Heterogenous study population (not exclusively SCI patients)	1[24]
Metastatic SC compression	1[25]
Neurological condition (amyotrophic lateral sclerosis)	1[26]
No association between imaging and clinical outcome	1[27]

Note: PubMed.gov search with the terms “prospective, acute spinal cord injury, magnetic resonance imaging, complications”

References S1.

1. Giannarini G, Kessler TM, Roth B, Vermathen P, Thoeny HC. Functional multiparametric magnetic resonance imaging of the kidneys using blood oxygen level dependent and diffusion-weighted sequences. *J Urol*. Aug 2014;192(2):434-9. doi:10.1016/j.juro.2014.02.048
2. Katsumi K, Yamazaki A, Watanabe K, Ohashi M, Shoji H. Can prophylactic bilateral C4/C5 foraminotomy prevent postoperative C5 palsy after open-door laminoplasty?: a prospective study. *Spine (Phila Pa 1976)*. Apr 2012;37(9):748-54. doi:10.1097/BRS.0b013e3182326957
3. Ichihara D, Okada E, Chiba K, et al. Longitudinal magnetic resonance imaging study on whiplash injury patients: minimum 10-year follow-up. *J Orthop Sci*. Sep 2009;14(5):602-10. doi:10.1007/s00776-009-1378-z
4. Marquardt G, Setzer M, Szelenyi A, Seifert V, Gerlach R. Significance of serial S100b and NSE serum measurements in surgically treated patients with spondylotic cervical myelopathy. *Acta Neurochir (Wien)*. Nov 2009;151(11):1439-43. doi:10.1007/s00701-009-0408-0
5. Como JJ, Thompson MA, Anderson JS, et al. Is magnetic resonance imaging essential in clearing the cervical spine in obtunded patients with blunt trauma? *J Trauma*. Sep 2007;63(3):544-9. doi:10.1097/TA.0b013e31812e51ae
6. Summers B, Malhan K, Cassar-Pullicino V. Low back pain on passive straight leg raising: the anterior theca as a source of pain. *Spine (Phila Pa 1976)*. Feb 2005;30(3):342-5. doi:10.1097/01.brs.0000152378.93868.c8
7. Ishibe T, Takahashi S. Respiratory dysfunction in patients with chronic-onset cervical myelopathy. *Spine (Phila Pa 1976)*. Oct 2002;27(20):2234-9. doi:10.1097/00007632-200210150-00010
8. Friedman D, Flanders A, Thomas C, Millar W. Vertebral artery injury after acute cervical spine trauma: rate of occurrence as detected by MR angiography and assessment of clinical consequences. *AJR Am J Roentgenol*. Feb 1995;164(2):443-7; discussion 448-9. doi:10.2214/ajr.164.2.7839986

9. Tosi L, Righetti C, Terrini G, Zanette G. Atypical syndromes caudal to the injury site in patients following spinal cord injury. A clinical, neurophysiological and MRI study. *Paraplegia*. Nov 1993;31(11):751-6. doi:10.1038/sc.1993.117
10. Bush L, Brookshire R, Roche B, et al. Evaluation of Cervical Spine Clearance by Computed Tomographic Scan Alone in Intoxicated Patients With Blunt Trauma. *JAMA Surg*. 09 2016;151(9):807-13. doi:10.1001/jamasurg.2016.1248
11. Arija-Blázquez A, Ceruelo-Abajo S, Díaz-Merino MS, et al. Effects of electromyostimulation on muscle and bone in men with acute traumatic spinal cord injury: A randomized clinical trial. *J Spinal Cord Med*. May 2014;37(3):299-309. doi:10.1179/2045772313Y.0000000142
12. Sabre L, Tomberg T, Kõrv J, et al. Brain activation in the acute phase of traumatic spinal cord injury. *Spinal Cord*. Aug 2013;51(8):623-9. doi:10.1038/sc.2013.41
13. Kim K, Mishina M, Kokubo R, et al. Ketamine for acute neuropathic pain in patients with spinal cord injury. *J Clin Neurosci*. Jun 2013;20(6):804-7. doi:10.1016/j.jocn.2012.07.009
14. Kelly JC, O'Briain DE, Kelly GA, Mc Cabe JP. Imaging the spine for tumour and trauma--a national audit of practice in Irish hospitals. *Surgeon*. Apr 2012;10(2):80-3. doi:10.1016/j.surge.2011.01.007
15. Hiersemenzel LP, Curt A, Dietz V. From spinal shock to spasticity: neuronal adaptations to a spinal cord injury. *Neurology*. Apr 2000;54(8):1574-82. doi:10.1212/wnl.54.8.1574
16. Wang-Leandro A, Hobert MK, Alisauskaite N, et al. Spontaneous acute and chronic spinal cord injuries in paraplegic dogs: a comparative study of in vivo diffusion tensor imaging. *Spinal Cord*. 12 2017;55(12):1108-1116. doi:10.1038/sc.2017.83
17. Wang-Leandro A, Siedenburg JS, Hobert MK, et al. Comparison of Preoperative Quantitative Magnetic Resonance Imaging and Clinical Assessment of Deep Pain Perception as Prognostic Tools for Early Recovery of Motor Function in Paraplegic Dogs with Intervertebral Disk Herniations. *J Vet Intern Med*. May 2017;31(3):842-848. doi:10.1111/jvim.14715
18. Forterre F, Gorgas D, Dickomeit M, Jaggy A, Lang J, Spreng D. Incidence of spinal compressive lesions in chondrodystrophic dogs with abnormal recovery after hemilaminectomy for treatment of thoracolumbar disc disease: a prospective magnetic resonance imaging study. *Vet Surg*. Feb 2010;39(2):165-72. doi:10.1111/j.1532-950X.2009.00633.x
19. Nout YS, Mihai G, Tovar CA, Schmalbrock P, Bresnahan JC, Beattie MS. Hypertonic saline attenuates cord swelling and edema in experimental spinal cord injury: a study utilizing magnetic resonance imaging. *Crit Care Med*. Jul 2009;37(7):2160-6. doi:10.1097/CCM.0b013e3181a05d41
20. Liu HM, Dong C, Zhang YZ, et al. Clinical and imaging features of spinal cord type of neuro Behçet disease: A case report and systematic review. *Medicine (Baltimore)*. Oct 2017;96(40):e7958. doi:10.1097/MD.0000000000007958
21. Takahata S, Shirado O, Minami A, Oda H. Quadriparesis due to acute collapse of a seemingly stabilized C5/6 segment in a patient with rheumatoid arthritis--a case report. *Orthopedics*. 04 2008;31(4):401. doi:10.3928/01477447-20080401-37
22. Tolonen A, Turkka J, Salonen O, Ahoniemi E, Alaranta H. Traumatic brain injury is under-diagnosed in patients with spinal cord injury. *J Rehabil Med*. Oct 2007;39(8):622-6. doi:10.2340/16501977-0101
23. Hadjipavlou A, Tosounidis T, Gaitanis I, Kakavelakis K, Katonis P. Balloon kyphoplasty as a single or as an adjunct procedure for the management of symptomatic vertebral haemangiomas. *J Bone Joint Surg Br*. Apr 2007;89(4):495-502. doi:10.1302/0301-620X.89B4.18121
24. Awad BI, Carmody MA, Lubelski D, et al. Adjacent Level Ligamentous Injury Associated with Traumatic Cervical Spine Fractures: Indications for Imaging and Implications for Treatment. *World Neurosurg*. Jul 2015;84(1):69-75. doi:10.1016/j.wneu.2015.02.029

25. McGivern UM, Drinkwater KJ, Clarke JI, Locke I. A royal college of radiologists national audit of radiotherapy in the treatment of metastatic spinal cord compression and implications for the development of acute oncology services. *Clin Oncol (R Coll Radiol)*. Aug 2014;26(8):453-60. doi:10.1016/j.clon.2014.04.032
26. García Santos JM, Blanquer M, Torres del Río S, et al. Acute and chronic MRI changes in the spine and spinal cord after surgical stem cell grafting in patients with definite amyotrophic lateral sclerosis: post-infusion injuries are unrelated with clinical impairment. *Magn Reson Imaging*. Oct 2013;31(8):1298-308. doi:10.1016/j.mri.2013.05.006
27. Lamothe G, Muller F, Vital JM, Goossens D, Barat M. Evolution of spinal cord injuries due to cervical canal stenosis without radiographic evidence of trauma (SCIWORET): a prospective study. *Ann Phys Rehabil Med*. Jun 2011;54(4):213-24. doi:10.1016/j.rehab.2011.02.003
